# Analysis of Risk Factors for Post-ERCP Pancreatitis in Patients with Cholangiocarcinoma

**DOI:** 10.3390/diagnostics16060900

**Published:** 2026-03-18

**Authors:** Takeshi Iizuka, Yusuke Kurita, Yu Honda, Takayuki Oda, Shin Yagi, Sho Hasegawa, Takamitsu Sato, Kunihiro Hosono, Noritoshi Kobayashi, Itaru Endo, Kensuke Kubota, Masato Yoneda

**Affiliations:** 1 Department of Gastroenterology and Hepatology, Yokohama City University Hospital, Yokohama 236-0004, Kanagawa, Japan; iizuka.tak.ax@yokohama-cu.ac.jp (T.I.); honda.yu.cy@yokohama-cu.ac.jp (Y.H.); t246016b@yokohama-cu.ac.jp (T.O.); t216074f@yokohama-cu.ac.jp (S.Y.); t166064d@yokohama-cu.ac.jp (S.H.); tkmtsato@yokohama-cu.ac.jp (T.S.); hiro1017@yokohama-cu.ac.jp (K.H.); kubotak@yokohama-cu.ac.jp (K.K.); yoneda@yokohama-cu.ac.jp (M.Y.); 2 Department of Oncology, Yokohama City University Hospital, Yokohama 236-0004, Kanagawa, Japan; norikoba@yokohama-cu.ac.jp; 3 Department of Gastroenterological Surgery, Yokohama City University Hospital, Yokohama 236-0004, Kanagawa, Japan; endoit@yokohama-cu.ac.jp

**Keywords:** endoscopic retrograde cholangiopancreatography, post-ERCP pancreatitis, cholangiocarcinoma, risk factors, outcomes

## Abstract

**Background/Objectives:** Endoscopic retrograde cholangiopancreatography (ERCP) is commonly performed for cholangiocarcinoma and often involves multiple procedures, potentially increasing the risk of post-ERCP pancreatitis (PEP). However, PEP characteristics in cholangiocarcinoma patients remain unclear. This study aimed to assess the incidence and diagnostic procedure-specific risk factors for PEP in patients with cholangiocarcinoma. **Methods:** We retrospectively reviewed 218 ERCP procedures for cholangiocarcinoma performed at our hospital between January 2017 and March 2022. The incidence of PEP, severe PEP, and fatal PEP was recorded. Risk factors for PEP were analyzed using multivariate analysis, and severe cases were further evaluated. **Results:** Among the 218 patients, 15 (6.9%) developed PEP, 4 (1.8%) had severe PEP, and 2 (0.9%) died. Multivariate analysis identified the pancreatic guidewire technique (PGW) (OR: 8.18; 95% CI: 2.52–26.53, *p* < 0.001) and intraductal ultrasonography (IDUS) (OR: 6.53; 95% CI: 2.01–21.25, *p* = 0.002) as significant risk factors. Both fatal cases involved naïve papilla and PGW and IDUS, with no pancreatic duct stent placement. **Conclusions:** ERCP for cholangiocarcinoma carries a clinically relevant risk of severe or fatal PEP. In particular, ERCP-specific diagnostic procedures required for cholangiocarcinoma may confer a disease-specific risk profile distinct from that of general ERCP. PGW and IDUS were identified as major risk factors, especially in patients with a naïve papilla, in whom prophylactic pancreatic duct stenting should be considered to reduce the risk of severe or fatal outcomes.

## 1. Introduction

Endoscopic retrograde cholangiopancreatography (ERCP) is essential for the diagnosis and treatment of biliary and pancreatic diseases [1]. However, ERCP has the potential to cause various adverse events, including post-ERCP pancreatitis (PEP) [2,3]. Pancreatitis is problematic because, once severe, it can require intensive care management and prolonged hospitalization (months to years), and it can even be a fatal complication [4]. The mechanisms of pancreatitis after ERCP include fluid stasis due to papillary edema, resulting in increased intraductal pressure; pancreatic duct injury due to the use of various devices; increased intraductal pressure due to frequent pancreatography, reflux in the pancreatic duct, water use, intraductal injection of contrast media or intestinal fluid, and intraductal papillary edema; and thermal injury to the pancreas due to the use of radiofrequency [4,5]. Recent systematic surveys have reported the incidence of PEP to be 3.5–10%, with severe cases accounting for 0.4–0.5% and fatalities accounting for 0.1–0.7% of patients who underwent ERCP [6,7].

In patients with cholangiocarcinoma, ERCP is necessary for diagnostic purposes, such as histological diagnosis and evaluation of the extent of disease using direct cholangiography, as well as for therapeutic purposes, such as the improvement of jaundice [8]. Cholangiocarcinoma requires different diagnostic and therapeutic approaches—depending on the site of origin—and has a tendency to spread horizontally, making the diagnosis of both benign and malignant lesions, as well as the extent of lesion extension, critical [9]. ERCP for cholangiocarcinoma may require additional investigations such as step biopsy, intraductal ultrasonography (IDUS), and oral cholangioscopy [10]. Therefore, ERCP for cholangiocarcinoma may be invasive and increase the risk of developing PEP. However, the details regarding PEP in patients with cholangiocarcinoma remain unclear. Therefore, this study aimed to determine the incidence rates of PEP, severe PEP, and fatal PEP in patients with cholangiocarcinoma and investigate the incidence, severity, and diagnostic procedure-specific risk factors for PEP development in patients with cholangiocarcinoma.

## 2. Methods

In this study, we collected data from 218 patients who underwent ERCP for cholangiocarcinoma at our hospital between January 2017 and March 2022. Because this study was a retrospective observational study, only preexisting medical records were reviewed, and no new patient information was collected. The present study was approved by the Institutional Review Board of our institution (approval number F241000004). The requirement for written informed consent was waived due to the retrospective nature of the study, and an opt-out consent process was implemented in accordance with institutional regulations.

### 2.1. ERCP Procedure

Cholangiocarcinoma was initially suspected based on cross-sectional imaging, most commonly magnetic resonance cholangiopancreatography (MRCP) or contrast-enhanced computed tomography.

All procedures were primarily performed under conscious sedation using intravenous midazolam and/or diazepam in combination with pentazocine, with continuous cardiorespiratory monitoring. Midazolam 2–10 mg, diazepam 5 mg, and pentazocine 15 mg were also administered intravenously. ERCP was performed under sedation or anesthesia.

All ERCP procedures were performed using a duodenoscope with a 15° backward-oblique viewing angle (TJF-Q290V, TJF-260V, or JF-260V; Olympus Medical Systems, Tokyo, Japan). Wire-guided cannulation (WGC) was used in all cases, and contrast-assisted cannulation was not performed during the study period. Conventional catheters (MTW; MTW Endoskopie, Dusseldorf, Germany, or SHOREN; Kaneka Corporation, Osaka, Japan) and guidewires (VisiGlide2; Olympus Medical Systems) were used. When selective biliary cannulation was difficult despite WGC, the pancreatic guidewire technique (PGW) was applied. In PGW cases, pancreatic duct stenting was principally intended for PEP prophylaxis but was performed selectively in patients considered at high risk. A 5-Fr, 3-cm single-flap plastic stent was used. However, stent placement was not possible in some PGW cases due to spontaneous guidewire dislodgement, inability to advance the stent into the pancreatic duct, or unstable scope position. These situations explain why only 21 of 47 PGW procedures resulted in pancreatic stent placement. However, in cases where the guidewire could not be placed in the pancreatic duct, a precut sphincterotomy was performed. A papillotomy knife (CleverCut3V; Olympus Medical Systems) was used for endoscopic sphincterotomy (EST), which was performed unless patients were receiving antithrombotic or anticoagulant therapy. In principle, IDUS was performed before EST to evaluate the longitudinal tumor extent in naïve papilla cases requiring detailed preoperative assessment. Bile duct biopsies were performed after EST when required. An IDUS (UM-G20-29R; Olympus, Tokyo, Japan) was inserted into the bile duct over the guidewire, and biopsy forceps (Radial Jaw 4 Pediatric; Boston Scientific Japan, Tokyo, Japan, or wire-guided one-sided opening-cup biopsy forceps; Olympus, Tokyo, Japan) were used to conduct bile duct biopsies.

As drainage, a bile duct stent was placed at the end of the procedure. The endoscopist determined the type of stent. The stents used were transpapillary, fully covered metal or plastic stents. The plastic stents of choice were an endoscopic nasobiliary drainage (ENBD) tube, an inside stent, and a transpapillary stent. As a general policy, pancreatic duct stents were placed in PGW cases whenever technically feasible. During ERCP, intraductal ultrasonography (IDUS) was selectively performed to evaluate longitudinal tumor extension and ductal wall invasion when clinically indicated. Peroral cholangioscopy was not routinely performed during the initial ERCP, according to our institutional diagnostic strategy.

Rectal NSAIDs and protease inhibitors were not administered at our institution during the study period, and no standardized prophylactic hydration protocol for PEP prevention was adopted.

### 2.2. Definition of PEP and Adverse Events

PEP was diagnosed according to the following criteria: new-onset upper abdominal pain and at least a three-fold increase in pancreatic enzyme levels within 24 h post-ERCP and on the second day of hospitalization [11,12]. The severity of pancreatitis was defined based on the Cotton classification [13], and other procedure-related adverse events were defined based on the severity grading system presented in the American Society for Gastrointestinal Endoscopy lexicon [12].

### 2.3. Endpoints

The primary endpoint of this study was the incidence of PEP in patients with cholangiocarcinoma. The secondary endpoints were the incidence rates of severe and fatal PEP. Thus, factors influencing PEP development and details regarding severe or fatal PEP were evaluated.

### 2.4. Statistical Analysis

All statistical analyses were performed using IBM SPSS Statistics (version 29.0; IBM Corp., Armonk, NY, USA), with statistical significance set at *p* < 0.05. Continuous variables are presented as medians (range), and categorical variables as frequencies (percentages). The Mann–Whitney U test was used for comparisons of continuous variables, while Pearson’s χ^2^ test or Fisher’s exact test was used for categorical variables. Variables associated with PEP in univariate analysis (*p* < 0.05) were considered for multivariable logistic regression analysis. Given the limited number of PEP events (*n* = 15) relative to the overall sample size, the number of variables included in the multivariable model was restricted based on event-per-variable considerations to minimize the risk of overfitting, and two clinically relevant procedure-related variables were selected a priori.

## 3. Results

### 3.1. Study Population

In total, 218 patients with cholangiocarcinoma treated with ERCP performed for the first time at our hospital were evaluated in this study. As shown in Table 1, which presents the patient background characteristics, the patients had a median age of 71.5 (31.0–91.0) years, 59 (27.0%) were female, 108 (49.5%) had naïve papilla, and 52 (23.8%) were treated for more than 60 min. The disease breakdown was as follows: 38 cases of distal cholangiocarcinoma, 152 cases of hilar cholangiocarcinoma, and 28 cases of intrahepatic cholangiocarcinoma. Although this study included only patients undergoing their first ERCP at our institution, some patients had undergone ERCP at previous hospitals; therefore, the proportion of naïve papilla cases was less than half of the cohort.

### 3.2. Incidence of PEP and Severe PEP, Mortality Rate, and Other Complications

**Figure 1:**
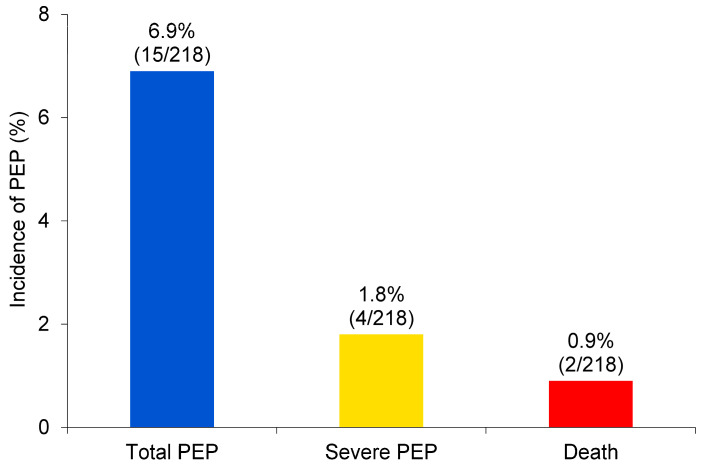
The incidence of PEP, severe PEP, and fatal PEP in patients with cholangiocarcinoma. Abbreviations: PEP, post-endoscopic retrograde cholangiopancreatography pancreatitis.

The incidence rates of PEP, severe PEP, and mortality in the overall cohort were 6.9% (15/218), 1.8% (4/218), and 0.9% (2/218), respectively (Figure 1). Other complications included bleeding in 0.4% of patients (1/218), cholangitis in 0.9% (2/218), and perforation in 0.0% (0/218) (Table 2). Cholangitis improved with conservative treatment. For the bleeding case, endoscopy was performed the next day, endoscopic balloon compression was performed, and the stent was replaced with a fully covered metal stent.

### 3.3. Factors Influencing the Development of PEP

Univariate analysis revealed that the following factors were significantly associated with the development of PEP: ERCP procedure time (>60 min; *p* = 0.001), EST (*p* < 0.001), pancreatic duct stent (*p* < 0.001), IDUS (*p* < 0.001), pancreatography (*p* < 0.001), and the PGW (*p* < 0.001) (Table 3). Given the limited number of PEP events (*n* = 15), multivariable logistic regression analysis was restricted to two clinically relevant and procedure-related variables (PGW and IDUS) to minimize overfitting. In this model, use of PGW (odds ratio [OR]: 8.18; 95% CI: 2.52–26.53, *p* < 0.001) and IDUS (OR: 6.53; 95% CI: 2.01–21.25, *p* = 0.002) remained independently associated with the development of PEP.

### 3.4. Details Regarding PEP in the Severe and Fatal Cases

**Figure 2:**
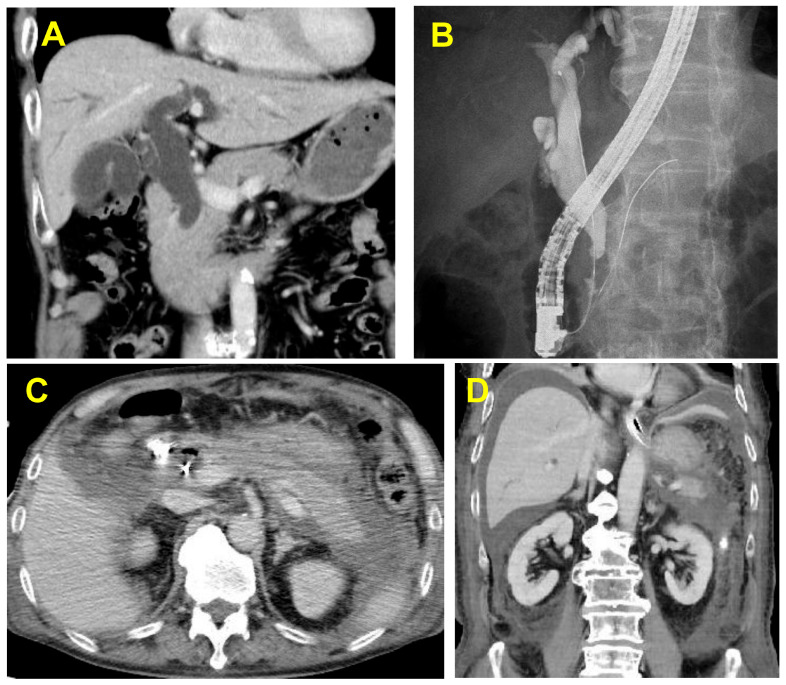
Images from a case of fatal PEP in a male patient with distal cholangiocarcinoma (case 1 in Table 4): (**A**) Contrast-enhanced CT revealing a tumor with contrast effect on the distal bile duct and bile duct dilatation upstream of the stenosis. (**B**) ERCP revealing distal bile duct stenosis due to distal cholangiocarcinoma. The patient was revealed to have a naïve papilla and underwent bile duct cannulation using the PGW, IDUS, and bile duct biopsies. (**C**) Enhanced CT performed on the day after ERCP showing an increased peripancreatic lipid concentration, appearance of a contrast-ineffective zone over the entire pancreas, and inflammatory spillover to the subrenal pole and beyond, with the patient experiencing abdominal pain and elevated pancreatic enzyme levels. The patient later developed respiratory and renal failure, was placed on a ventilator, and underwent continuous dialysis in the intensive care unit. (**D**) Contrast-enhanced CT performed 1 week after ERCP revealing marked signs of inflammation and abscess formation in the abdominal cavity. Percutaneous intraperitoneal abscess drainage was added, but there was little improvement. Two months after ERCP, drainage surgery was performed to treat the intra-abdominal abscess. Thereafter, there was little improvement, and the patient died 3 months after ERCP. This case illustrates the potential lethality of PEP following invasive ERCP procedures requiring PGW and IDUS in a naïve papilla. Abbreviations: PEP, post-endoscopic retrograde cholangiopancreatography; CT, computed tomography; ERCP, endoscopic retrograde cholangiopancreatography; IDUS, intraductal ultrasonography; PGW, pancreatic guidewire technique.

Table 4 presents the details of the severe and fatal cases of PEP. IDUS was also performed in three of the four cases. In the two fatal cases, naïve papilla was involved; bile duct cannulation was performed using PGW; and IDUS, EST, and bile duct biopsies were performed. However, no pancreatic duct stents were implanted. The clinical course of a fatal case of PEP is shown in Figure 2.

### 3.5. Temporal Trends in PEP Incidence

To explore potential temporal trends, we compared the early period (2017–2019) with the later period (2020–2022). The incidence of PEP was 8.5% (10/118) in 2017–2019 and 5.0% (5/100) in 2020–2022, with no statistically significant difference between the two periods (*p* = 0.313).

## 4. Discussion

In this study, we found that the incidence of PEP after ERCP for cholangiocarcinoma was 6.9% (15/218), with severe PEP occurring in 1.8% (4/218) and mortality in 0.9% (2/218) of patients. Although the present study did not include a control group of patients undergoing ERCP for other indications, precluding direct comparison with the general ERCP population, the observed rates of severe and fatal PEP (1.8% and 0.9%, respectively) appear numerically higher than those reported in recent systematic surveys (0.4–0.5% for severe cases and 0.1–0.7% for mortality) [6,7]. Therefore, while it cannot be definitively concluded that cholangiocarcinoma itself confers a higher risk, ERCP performed for cholangiocarcinoma may be associated with a relatively increased incidence of severe and potentially fatal PEP; however, this possibility should be interpreted with caution, given the absence of a control group.

The present study also identified the use of the PGW and IDUS as risk factors for PEP development in patients who undergo ERCP for cholangiocarcinoma. The PGW remained an independent risk factor for PEP. Although PGW facilitates biliary cannulation, it inevitably increases manipulation within the pancreatic duct and often reflects technically challenging cases. In addition, in some PGW cases, pancreatic duct stenting could not be performed despite intention, possibly increasing the susceptibility to PEP. IDUS is an important diagnostic modality for evaluating longitudinal tumor extension in cholangiocarcinoma; however, its impact on post-ERCP pancreatitis risk has not been sufficiently evaluated. IDUS was identified as an independent risk factor for PEP in this study. At our institution, IDUS is selectively performed when assessment of tumor extension is expected to influence treatment strategy. In principle, IDUS is performed before EST to allow detailed evaluation under relatively preserved papillary anatomy, after which EST is carried out when therapeutically indicated. Given that IDUS was generally performed before EST in cases requiring detailed tumor assessment, the procedure may have caused temporary outflow obstruction of pancreatic juice when performed in the setting of a naïve papilla.

Previous studies have demonstrated that prophylactic pancreatic duct stenting reduces the incidence of PEP [14]. Additionally, EST has been reported to decrease the risk of PEP in patients with biliary neoplasms [15]. In our univariate analysis, however, EST and pancreatic duct stenting were significantly associated with the occurrence of PEP. This finding contradicts previous evidence showing their preventive effects; however, this paradox is likely due to selection bias. Both interventions were performed predominantly in cases where bile duct cannulation was particularly difficult—conditions that inherently increase PEP risk. Therefore, the association observed in univariate analysis should not be interpreted as a causal relationship but rather as a reflection of procedural complexity and risk-based patient selection. The higher incidence of PEP observed in patients undergoing EST in this study may reflect case complexity rather than a direct harmful effect of EST itself. In our cohort, EST was more frequently performed in patients with a naïve papilla and in those requiring additional invasive diagnostic procedures, such as IDUS and bile duct biopsy. These factors likely contributed to increased procedural invasiveness and may have collectively elevated the risk of PEP.

Although various risk factors for PEP have been reported, the specific risk profile in patients undergoing ERCP for cholangiocarcinoma remains incompletely defined. In the general population, known patient-related risk factors include sphincter of Oddi dysfunction, younger age, female sex, prior PEP, and naïve papilla [16,17], while procedural factors such as precut sphincterotomy, repeated pancreatography, and the PGW are also established contributors [18,19]. In the present study, IDUS was identified as a risk factor for PEP development. Generally, IDUS can be performed under guidewire guidance during ERCP [20]. In patients undergoing ERCP for cholangiocarcinoma, IDUS can be used to assess the tumor’s extension and extramural depth [21,22]. Although EST has previously been reported to reduce the incidence of PEP in patients with cholangiocarcinoma [15], evaluating the tumor’s extension and extramural depth using IDUS after EST might be more difficult due to the increased entry of intestinal gas. Therefore, although IDUS may be considered after EST to reduce pancreatitis, it may also prevent the evaluation of tumor localization in the biliary tract; however, further studies are required to investigate the associations between these factors. Accordingly, IDUS should be applied selectively rather than routinely, particularly in cases requiring technically demanding cannulation. At our institution, peroral cholangioscopy was reserved for selected cases rather than routinely performed during the initial ERCP; therefore, IDUS represented the primary intraductal diagnostic modality during the study period.

We also identified the PGW as a risk factor for PEP development in this study. The PGW is a method used to obtain bile duct cannulation in difficult cases by placing a guidewire in the pancreatic duct [18,23]. Thus, this method might place more stress on the pancreas and increase the risk of developing pancreatitis [24]. In contrast, pancreatic duct stenting has previously been shown to reduce the risk of PEP [25,26]; however, the use of pancreatic duct stents did not reduce the risk of pancreatitis in the present study. This may have been because the cases in which pancreatic duct stents were placed were those in which bile duct intubation was performed using the PGW, and there were many cases in which bile duct intubation was difficult, increasing the risk for PEP.

All severe or fatal PEP cases involved naïve papilla and prolonged cannulation attempts requiring PGW and IDUS. Importantly, no pancreatic duct stent was placed in the two fatal cases. These observations highlight the need to consider prophylactic pancreatic stenting or, alternatively, to avoid additional invasive diagnostic procedures when PGW is required in naïve papilla cases. In this study, severe PEP was developed in four patients, two of whom died. All four patients had naïve papilla, and the PGW and IDUS were performed in all but one case. In both cases that resulted in death, naïve papilla was present, and IDUS, EST, and bile duct biopsy were performed, but no pancreatic duct stent was placed. Therefore, if an invasive ERCP examination—such as the one described above—is performed and a pancreatic duct stent cannot be placed, the possibility of developing severe, and even fatal, PEP exists. In such cases, it may be better to take certain measures to prevent pancreatitis to the greatest extent possible, such as administering NSAIDs [11], performing fluid therapy [27,28,29], and ensuring that a pancreatic duct stent is placed when using the PGW to intubate the bile duct. Moreover, in cases where bile duct cannulation is difficult, such as when performing the PGW on a naïve papilla, or where there are other risk factors for PEP, it may be preferable to avoid additional invasive examinations such as bile duct biopsy or IDUS.

From a clinical perspective, our findings suggest that ERCP strategies for cholangiocarcinoma should differ from those for other biliary diseases. In particular, when difficult biliary cannulation requires the PGW in patients with a naïve papilla, additional invasive diagnostic procedures, such as IDUS or bile duct biopsy, should be carefully reconsidered. If these procedures are unavoidable, prophylactic pancreatic duct stenting and other preventive measures should be strongly considered.

We did not observe a statistically significant temporal difference in PEP incidence between the early (2017–2019) and later (2020–2022) study periods. Although incremental refinements in ERCP techniques and increasing operator experience over time might have influenced procedural safety, our data did not demonstrate a measurable reduction in PEP incidence during the study period. Nevertheless, subtle improvements cannot be entirely excluded given the limited number of events.

This study had some limitations. First, it was based on data collected from a single center. Second, this was a retrospective, observational study. Because only 15 PEP events occurred, the number of variables that could be reliably included in the multivariable model was limited. Therefore, only two clinically relevant variables were included to minimize overfitting. Third, rectal NSAIDs and aggressive periprocedural hydration were not routinely used during the study period, which may limit the generalizability of our findings to contemporary clinical practice. As these measures are now widely recommended for PEP prophylaxis [11,27,28,29], the incidence and severity observed in this cohort may not directly reflect outcomes under current preventive strategies. Fourth, changes in ERCP techniques and prophylactic strategies over time may limit the applicability of our findings to current practice. Fifth, we did not evaluate the potential impact of different sedative agents on PEP occurrence, as the primary focus of this study was on procedural risk factors.

## 5. Conclusions

In conclusion, ERCP for cholangiocarcinoma carries a clinically relevant risk of severe or fatal PEP. The PGW and IDUS were identified as significant procedure-related risk factors in this cohort. Notably, the fatal cases involved naïve papilla and required PGW and IDUS without successful pancreatic duct stenting. These findings underscore the importance of careful procedural planning and consideration of prophylactic strategies when performing complex diagnostic ERCP in patients with cholangiocarcinoma.

## Figures and Tables

**Table 1:** Baseline patient characteristics.

Characteristics	*n* = 218
Age, years	71.5 (31.0–91.0)
Sex, female	59 (27.0)
Tumor location (cholangiocarcinoma)	
Distal	38 (17.4)
Hilar	152 (69.7)
Intrahepatic	28 (12.8)
ERCP procedure time, min	43.8 (7.0–120.0)
Procedure time >60 min	52 (23.8)
Naïve papilla	108 (49.5)
Pancreatography	56 (25.6)
PGW	47 (21.5)
Precut sphincterotomy	5 (2.3)
IDUS	54 (24.7)
EST	78 (35.7)
Bile duct biopsy	130 (59.6)
Bile duct cytology	84 (38.5)
Biliary drainage stent ^†^	
ENBD	38 (17.4)
Inside PS	44 (20.2)
Transpapillary PS	141 (64.7)
Transpapillary fully covered MS	7 (3.2)
Pancreatic duct stent	21 (9.6)
Period	
2017–2019	118 (54.1)
2020–2022	100 (45.9)

Data are presented as the median (range) and *n* (%). Abbreviations: ERCP, endoscopic retrograde cholangiopancreatography; EST, endoscopic sphincterotomy; IDUS, intraductal ultrasonography; PGW, pancreatic guidewire technique; ENBD, endoscopic nasobiliary drainage; PS, plastic stent; MS, metal stent. ^†^ Percentages exceed 100% because multiple stent types were used in some patients.

**Table 2:** Complications of ERCP.

Complications	*n* = 218
PEP (total)	15 (6.9)
Severe PEP	4 (1.8)
Fatal PEP	2 (0.9)
Bleeding	1 (0.4)
Cholangitis	2 (0.9)
Perforation	0 (0.0)

Data are presented as *n* (%). Abbreviations: ERCP, endoscopic retrograde cholangiopancreatography; PEP, post-ERCP pancreatitis.

**Table 3:** Univariate and multivariate analysis of factors related to PEP.

Factor		*n*	PEP (%)	Univariate *p*-Value	Multivariate *p*-Value ^†^	OR (95% CI)
Age	≤50 years	6	16.7	0.337		
	>50 years	212	6.6			
Sex	Female	59	10.2	0.243		
	Male	159	5.7			
Indication	Hilar region	180	7.8	0.255		
	Distal	38	2.6			
Procedure time >60 min	Yes	52	13.5	0.032		
	No	166	4.8			
Naïve papilla	Yes	108	10.2	0.056		
	No	110	3.6			
Pancreatography	Yes	56	16.1	0.002		
	No	162	3.7			
PGW	Yes	47	21.3	<0.001	<0.001	8.18 (2.52–26.53)
	No	171	2.9			
Precut sphincterotomy	Yes	5	0.0	0.539		
	No	213	7.0			
IDUS	Yes	54	18.5	<0.001	0.002	6.53 (2.01–21.25)
	No	164	3.0			
EST	Yes	78	15.4	<0.001		
	No	140	2.1			
Bile duct biopsy	Yes	130	9.2	0.096		
	No	88	3.4			
Bile duct cytology	Yes	84	10.7	0.077		
	No	134	4.5			
ENBD	Yes	38	5.3	0.496		
	No	180	7.2			
Inside PS	Yes	44	13.6	0.057		
	No	174	5.2			
Transpapillary PS	Yes	141	5.7	0.341		
	No	77	9.1			
Transpapillary fully covered MS	Yes	7	0	0.465		
	No	211	7.1			
Pancreatic duct stent	Yes	21	23.8	0.001		
	No	197	5.1			
Period	2017–2019	118	8.5	0.313		
	2020–2022	100	5.0			

Abbreviations: PEP, post-endoscopic retrograde cholangiopancreatography pancreatitis; OR, odds ratio; CI, confidence interval; PGW, pancreatic guidewire technique; IDUS, intraductal ultrasonography; EST, endoscopic sphincterotomy; ENBD, endoscopic nasobiliary drainage; PS, plastic stent; MS, metal stent. ^†^ Due to the limited number of PEP events, only PGW and IDUS were included in the multivariable model to minimize overfitting.

**Table 4:** Details of the cases with severe or fatal PEP.

Case	Outcome	Sex	Tumor Location	Procedural Time, min	Naïve Papilla	PGW	IDUS	EST	Bile Duct Biopsy	Pancreatic Duct Stent
1	Fatal	M	Distal	43	Yes	Yes	Yes	Yes	Yes	No
2	Fatal	F	Hilar	68	Yes	Yes	Yes	Yes	Yes	No
3	Severe	M	Hilar	59	Yes	Yes	Yes	Yes	No	Yes
4	Severe	F	Hilar	64	Yes	No	No	Yes	Yes	No

Abbreviations: PEP, post-endoscopic retrograde cholangiopancreatography pancreatitis; PGW, pancreatic guidewire technique; IDUS, intraductal ultrasonography; EST, endoscopic sphincterotomy; M, male; F, female.

## Data Availability

The data presented in this study are available from the corresponding author upon reasonable request. The data are not publicly available due to ethical and privacy restrictions.
